# Translational design for limited resource settings as demonstrated by Vent-Lock, a 3D-printed ventilator multiplexer

**DOI:** 10.1186/s41205-022-00148-6

**Published:** 2022-09-14

**Authors:** Helen Xun, Christopher Shallal, Justin Unger, Runhan Tao, Alberto Torres, Michael Vladimirov, Jenna Frye, Mohit Singhala, Brockett Horne, Bo Soo Kim, Broc Burke, Michael Montana, Michael Talcott, Bradford Winters, Margaret Frisella, Bradley S. Kushner, Justin M. Sacks, James K. Guest, Sung Hoon Kang, Julie Caffrey

**Affiliations:** 1 Johns Hopkins School of Medicine, Baltimore, MD 21231 USA; 2 Department of Biomedical Engineering, Johns Hopkins University, Baltimore, MD 21231 USA; 3 Department of Civil and Systems Engineering, Johns Hopkins University, Baltimore, MD 21218 USA; 4 Maryland Institute College of Art, Baltimore, MD 21217 USA; 5 Department of Mechanical Engineering and Institute for NanoBioTechnology, Johns Hopkins University, Baltimore, MD 21218 USA; 6 Washington University in St. Louis School of Medicine, St. Louis, MO 63130 USA

**Keywords:** 3D printing, Vat photopolymerization, Material extrusion, Austere medicine, Covid-19, Limited resources, De novo system, Ventilator multiplexer, In vivo study

## Abstract

**Background:**

Mechanical ventilators are essential to patients who become critically ill with acute respiratory distress syndrome (ARDS), and shortages have been reported due to the novel severe acute respiratory syndrome coronavirus 2 (SARS-CoV-2).

**Methods:**

We utilized 3D printing (3DP) technology to rapidly prototype and test critical components for a novel ventilator multiplexer system, Vent-Lock, to split one ventilator or anesthesia gas machine between two patients. FloRest, a novel 3DP flow restrictor, provides clinicians control of tidal volumes and positive end expiratory pressure (PEEP), using the 3DP manometer adaptor to monitor pressures. We tested the ventilator splitter circuit in simulation centers between artificial lungs and used an anesthesia gas machine to successfully ventilate two swine.

**Results:**

As one of the first studies to demonstrate splitting one anesthesia gas machine between two swine, we present proof-of-concept of a de novo, closed, multiplexing system, with flow restriction for potential individualized patient therapy.

**Conclusions:**

While possible, due to the complexity, need for experienced operators, and associated risks, ventilator multiplexing should only be reserved for urgent situations with no other alternatives. Our report underscores the initial design and engineering considerations required for rapid medical device prototyping via 3D printing in limited resource environments, including considerations for design, material selection, production, and distribution. We note that optimization of engineering may minimize 3D printing production risks but may not address the inherent risks of the device or change its indications. Thus, our case report provides insights to inform future rapid prototyping of medical devices.

**Supplementary Information:**

The online version contains supplementary material available at 10.1186/s41205-022-00148-6.

## Background

Limited resource settings in healthcare result when demand outpaces supply, due to limits in time such as in emergency situations (e.g. Las Vegas shooting of 2019, war frontiers, natural disasters), demand (e.g. ventilator shortages during the coronavirus 2/SARS-CoV-2 pandemic), or supply (supply chain disruptions, materials shortages). The settings can be either acute (global pandemics), or chronic (developing countries). Limited resource settings pose unique challenges in solution design, manufacturing, and production of medical supplies. For example, the introduction of de novo or commercial products represents a theoretical ideal that cannot be achieved due to monetary, time, hospital infrastructure, limited scaling and production capacity, and supply chain constraints [1, 2], and lack of standardization of parts across manufacturers [3]. Solutions using off-the-shelf components may face limited or unreliable supply [4]. Even in developed countries with no existing deficit in supply, shortages may occur up to 6 months from the inciting events due to delays in supply chain [5]. Furthermore, these parts are often not optimized for, nor medical grade for medical applications. Consequently, in resource limited environments, rapid prototyping with additive manufacturing, although requiring more upfront work, is ultimately more sustainable.

3D printing has come to the forefront during the COVID-19 pandemic to address critical medical equipment shortages [3, 6], bridging gaps in supply shortages while traditional supply chains increase to meet demand [7, 8], and rapid prototyping to meet new needs [9–11]. 3D printing is cost-effective for small batches, with rapid on-demand production modality with broad applications due to its ability to produce intricate and complex geometries from computer-aided designs without tooling and expensive machines [12]. 3D printing enables faster design, prototyping, and manufacturing processes [13, 14] so that it can be utilized in limited resource settings to fill gaps in the supply chain [15]. However, bridging the gap between 3D printing and the medical industry has been challenging, as it requires individuals with extensive insight and experience into both, or multidisciplinary team collaboration. Engineering details and nuances can have direct impact on patient outcomes. For example, vat photopolymerization is often the preferred production method for medical devices compared to material extrusion, whose higher layer resolution often results in microscale air pockets that may become niduses for bacterial infection [14].

One application of 3D printing during the COVID-19 pandemic was to address ventilator shortages. Approximately 5–10% of patients with coronavirus disease (COVID-19) become critically ill from acute respiratory distress syndrome (ARDS) and require ventilators [16]. Despite the introduction of vaccines, variants of concern (VOCs: Alpha, Beta, Gamma, Delta, Omicron, Epsilon) continue to emerge. It is predicted that the current global COVID death toll of 5 million is a gross underestimate, and excess-deaths are not always reported. This acute demand further exacerbates chronically limited resources in developing countries, which has historically experienced higher mortality rates during pandemics [17]. For example, the continent of Africa has limited ventilator capacity, with only ~ 2000 ventilators across 41 countries [18]. Countries with fragile healthcare infrastructures also experience more COVID excess-deaths. For example, while India reports fewer than 500,000 COVID-19 deaths [19], the prediction is closer to 3 to 5 million people. The unreported excess-deaths that disproportionately affect developing countries demonstrate that in the setting of pandemics, treatment is resource limited, and even modern-day emerging solutions do not adequately address these disparities.

Ventilator shortages occur in resource-rich countries as well. To-date, the USA has the highest number of reported cases (81 million) and deaths (987,615) [20]. Despite the comparatively ample access to healthcare in the USA, projections suggest that hospitals may be operating at 120–160% capacity in the face of any given pandemic or national disaster [21]. The Society of Critical Care Medicine shared that clinicians reported ventilator shortages during the peak of the pandemic in Summer of 2020, including 53% of 587 surveyed ICU clinicians who did not have enough ventilators and had to use non-standard ventilators or non-invasive devices [22]. Consequently, in limited resource environments such as during COVID-19, there is a critical need to address unpredictable ventilator shortages.

An alternative strategy to quickly increase ventilator capacity is to “split” or multiplex ventilators and anesthesia gas machines, allowing for effective increases in capacity. The concept of using one ventilator to support multiple patients during a disaster surge was first published in 2006 [23], and demonstrated in actual patients during the 2017 Las Vegas shooting [24]. While this can significantly increase capacity, it is met with many clinical and engineering challenges.

Although possible, the clinical challenges of ventilator multiplexing have been well described. Emergency use of ventilator multiplexing is dependent on the dynamic lung states of the patients, including associated lung compliances and airway resistances that determine airflow balance. In the evolving pathologic state of COVID-19 patients with ARDS, an interdependent ventilation system poses many safety concerns. The Society of Critical Care Medicine and other societies in respiratory care issued a joint statement [25] summarizing main concerns with ventilator multiplexing [26], including the inability to independently monitor and control ventilation parameters (volumes, pressures, rates) critical for ARDS treatment, thus risking adverse outcomes. Additional concerns include ventilator alarm management, disrupted balance of ventilation if a patient has spontaneous breathing, sudden deterioration, kink in the tubing, and viral contamination if breathing circuits between patients are mixed, or the circuit becomes open. Therefore, while possible, it carries significant clinical risks.

Of particular interest for this article, and less commonly discussed among the critical care community, are the additional challenges introduced due to the de novo engineering (design and production) process. For example, computational fluid dynamics assessment of 3D printing demonstrates that, in theory, multi-patient ventilation with flow restricting orifices is possible. However, the flow resistance due to the rough interior surface of 3D printed restrictors and change in diameter can result in significant changes in resistance, and thus respiratory volumes and pressures [27]. In this study, we identify engineering translational considerations (design, production, materials, sterilization) when using 3D printing to rapidly prototype medical devices in limited resource settings.

We present our findings with the creation of Vent-Lock, a de novo*,* ventilator multiplexing system created at the start of the COVID-19 pandemic in the USA [28, 29]. Insights on clinical considerations were created by a multi-institutional, multi-disciplinary team. Vent-Lock can be rapidly produced via 3D printing, thus tapping into a broad international infrastructure largely unaffected by the pandemic [30–32]. We conducted simulation center and large animal trials to validate the use of Vent-Lock for ventilators and anesthesia gas machines which are more available in developing countries, to have more ventilation settings. Through this study, we share engineering considerations of de novo rapid medical device design, prototyping, and validation to the 3D printing community for future medical device production in limited resource environments.

## Methods

The aim of this study was 1) to identify challenges and considerations for prototyping and producing medical devices via 3D printing in limited resources such as during the COVID-19 pandemic and 2) to produce a ventilator splitting system to rapidly increase ventilator capacity. This study is a descriptive research article with proof-of-concept experimental lab and large mammal trials as described in the following sections. The setting was multi-institutional academia in two universities with engineering schools, and affiliated large teaching hospitals.

### Design committee

The design committee composed of clinicians (critical care physicians, anaesthesiologists, plastic surgeons, burn surgeons), engineers (mechanical, civil, materials engineers), clinical research scientists, graphic designers, 3D Printing artist, and FDA consultant. The translational team (clinicians, clinical research scientists, FDA consultant, lead engineers) met to identify challenges of 3DP in limited resource settings, design needs for a ventilator splitter, and rapid pre-clinical testing. The technical team (engineers, graphic designers, 3D Printing artist) met to optimize designs and production conditions for limited resource settings. The rapid prototyping cycle included production of the devices by the technical team, testing by the translational team with feedback to designs, followed by redesign and production by the technical team for improved generation of devices with each cycle. De novo ventilator circuit components are shown in Fig. S1.

### 3D printing procedure

3D printing of the Vent-Lock splitters, flow regulators, and manometer adaptors were produced via vat photopolymerization printers (Form 2, Form 3, or Form 3B, Formlabs, Somerville, MA) at 50 um layer resolutions, using surgical guide resin (Surgical Guide, Formlabs, Somerville, MA) and standard protocol per Formlabs [33]. Print files were generated by CAD drawings (SolidWorks, Dassault Systèms) and converted into G-code for 3D printing using the printer’s accompanying software package (PreForm, Formlabs, Somerville, MA). Support structures were minimized through design and generated using PreForm where needed. Components were oriented in such a way that crucial surfaces such as threads or O-ring ledges were not impacted by support structures. Prints were post-processed by washes (2 cycle with 15 min per cycle) in > 99.5% isopropyl alcohol (CAS Number: 67–63-0, Sigma Aldrich), followed by air-drying at 22 °C for 30 minutes, and post-cured for 30 minutes with heat 60 °C for the Form 2 printer and 70 °C for the Form 3B printer at 405 nm of light (Form Cure, Formlabs, Somerville, MA). O-rings (E1000–212/AS568–212, O-Rings EPDM, FDA EPDM, Marco Rubber & Plastics, Seabrook, New Hampshire, USA) were added for improved sealing. Production via material extrusion (e3d, BigBox3D Ltd., Oxfordshire, UK; Little Monster, Tevo 3D Electronic Technology Co. Ltd., Zhanjiang, China) used PETG Filament (PETG 3D Printer Filament, FilaMatrix, Virginia, USA). Print settings were a 0.2 mm layer height with 30% infill, nozzle temperature of 250 °C, and bed temperature of 70 °C; supports were generated from the build platform, with no interior supports. STL print files of the Vent-Lock splitter, FloRest, and manometer adaptor is available at Data S1.

### Sterilization testing

3D-printed parts produced from surgical guide resin were sterilized by dry vacuum autoclave (Sr 24C Adv-Plus™, Consolidated Sterilizer Systems, Boston, Massachusetts, USA), 3 cycles at 120.0 °C, 20 minutes sterilization time and 20 minutes dry time. Then, they were soaked in > 99.5% isopropyl alcohol (CAS Number: 67–63-0, Sigma Aldrich) for 30 minutes, air-dried at 22 °C for 30 minutes, and placed in an oven at 40 °C in humidified air for 48 hours (VO1824HPC, Lindberg/Blue M Vacuum Oven 127.4 L, Thermo Scientific, Waltham, MA, USA). Particle count analyses, which has been demonstrated in medical settings to correlate with biocontamination [34–36], were conducted using a particle counter (SOLAIR 3100, Lighthouse Worldwide Solutions), detecting sizes 0.3 to 10 μm, for 1-minute cycles, and performed for parts pre-autoclave, post-autoclave and post IPA wash, and humidified warm air exposure at 40 °C.

### Vent-Lock 1 + n(1) circuit and components

**Fig. 1:**
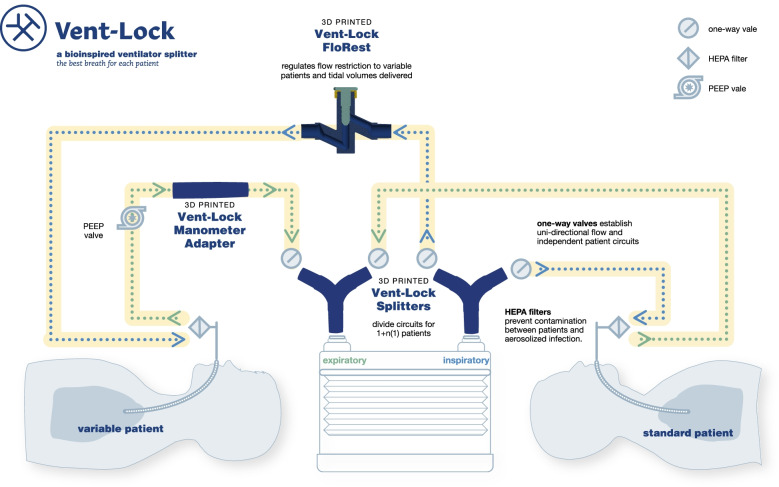
Vent-Lock ventilator multiplexing 1 + n(1) circuit and 3DP components. Our 1 + n(1) circuit proposes having a standard patient with minimal features, thus are ventilated per ventilator settings. Additional patients added to the circuit will be considered n(1), and will have variable flow and PEEP as controlled by circuit components. Please note that all patients, regardless of standard or variable, have one-way (check) valves and filters

Vent-Lock circuits were assembled as depicted in Fig. 1. Vent-Lock 3DP splitters, flow regulator, and manometer adaptors were used. Commercial components include manometer (Ambu Disposable Pressure Manometer, Ambu, Copenhagen, Denmark), one-way valves (22F × 22 M, REF 50245, Mallinckrodt Pharmaceuticals), disposable bacteria filters (BSF104, Vincent Medical), and ventilator tubing (SKU: 999027588, Hudson Rci). Air-tightness tests of the Vent-Lock FloRest is demonstrated in Fig. S2. Design files for all de novo components (splitters, needle valve, manometer adaptor) are available in Fig. S3.

### Simulation center testing

Vent-Lock 1 + n(1) circuits were tested at the Johns Hopkins Medicine Simulation Center (JHMSC). The ventilator (Puritan Bennett 840 Ventilator System, Avante Health Solutions) was set to pressure control mode of ventilation (Volume Ventilation Plus™, Avante Health Solutions) with additional settings detailed in Fig. S4. Vent-Lock 1 + 1 circuit was tested using test lungs simulating healthy lungs with variable compliances (Standard patient: R_p_ = 20 cmH_2_O/L/s, RespiTrainer Advance, QuickLung, IngMar Medical; Variable patient: R_p_ = 50 cmH_2_O/L/s, ASL 5000, IngMar Medical). Intrapulmonary data for both patients were collected; data included peak inspiratory pressures, tidal volumes, and peak end expiratory pressures. Five total values of tidal volume per data set were collected and averaged. Corresponding ventilator reported data was also recorded, including total expiratory volumes, peak inspiratory pressures, mean inspiratory pressures, and peak end expiratory pressures. Flow restrictors (#P20034 PVC SCH 40 ½-in FNPT Ball Valve; G300 Lead Free Brass Gate Valve; #P60SCPVC12 Stop and Waste Valve, American Valve, Greensboro, North Carolina, USA; Vent-Lock 3DP FloRest) were used to restrict the variable patient’s inspiratory flow rate per the 1 + n(1) circuit (Fig. 1). Valve handles were turned at smallest increments permissible to close the valve and documented as % closure. Corresponding intrapulmonary data and ventilator reported values were collected per handle closure and standardized to values (volumes and pressures) of a fully open valve (reported as proportion of maximum, %).

In vitro studies were also conducted at the Washington University Simulation Center using two Datex-Ohmeda Aestiva anesthesia machines. One machine was set to deliver pressure control ventilation in a manner similar to that performed at Johns Hopkins University. This machine was connected in parallel to a 2 L anesthesia bag reservoir and a second Datex-Ohmeda Aestiva machine that was set to spontaneous ventilation. The second machine served as a flow and volume sensor for the Vent-Lock 1 + n(1) circuit.

### In vivo swine studies

Experiments were performed in accordance with the Guide for the Care and Use of Laboratory Animals and were approved by the Institutional Animal Care and Use Committee of Washington University School of Medicine (St. Louis, MO). Domestic swine (*Sus scrofa domesticus*) were purchased from Oak Hill Genetics (Ewing, IL). The swine were females, 72 kg each, 5 months old, and were Landrace-cross swine. Swine were sedated with a telazol, ketamine, xylazine cocktail and intubated with a 7.0 endotracheal tube. Anesthesia was maintained with isoflurane. Femoral venous and arterial catheterization was performed. Standard ASA monitoring was maintained throughout the experiment. Swine were ventilated using a single ventilator (Drager Narkomed 2A) with two circuits in parallel in an 1 + n(1) configuration with cross-ventilation restricted by using one-way check valves. Ventilation was maintained with volume control. One swine was not flow-regulated and thus considered the standard patient, while the other had a Vent-Lock 3DP 4.0 connected in the inspiratory limb and thus considered the variable patient. Flow was measured at each expiatory limb with a SS11LB airflow transducer (Biopac; Goleta, CA). Flow data were collected at 2 kHz using an MP36 data acquisition unit and BSL 4.1.3 software (Biopac; Goleta, CA). The spirometry data was then smoothed with a 0.25 sec wide moving median filter after removal of instrument noise below 0.08 L/sec (determined by histogram inspection). The smoothed data was then numerically integrated to estimate respiratory tidal volume, and a first order numeric derivative was used to calculate the instantaneous respiratory rate. The noise floor for the integrated volume was determined by histogram inspection resulting in a threshold of 90 mL. The anesthesia record and the spirometry results were then aligned using common timestamps. All breaths spontaneously initiated by the swine (identified by respiratory rates more than 30% away from the ventilator set point) were removed from analysis. The mean and standard deviation for each anesthesia record entry were calculated for respiratory rate, tidal volume, minute ventilation, and lung compliance. All of the described analysis was performed using a custom MATLAB script (Data S2, MATLAB 2019b, The MathWorks, Inc., Natick, MA)]. Arterial and venous blood gas data were collected 15 minutes following any changes to the Vent-Lock 3DP device. Following the procedure, swine were euthanized with an overdose (~ 150 mg/kg) of supersaturated potassium chloride IV while under anesthesia. Necropsy was performed to assess for any gross lung pathology.

### Statistical analyses

Raw and calculated data were exported from MATLAB script (MATLAB 2019b, The MathWorks, Inc., Natick, MA). All statistical analyses were completed using Stata v.13 (StataCorp, College Station, TX) and Microsoft Excel (2018, Redmond, Washington). Variables were analyzed using two-tailed Student’s t tests and chi square analyses. The threshold for statistical significance was set at an alpha value of 0.05.

## Results

### Vent-Lock 1 + n(1) circuit and components

We validated a 1 + n(1) system which can split one ventilator between one standard patient and one or potentially more variable patients (Fig. 1). The standard patient ideally has the lowest lung compliance and has minimal components in the circuit to establish low resistance allowing the ventilator to maintain standard function. The standard patient will be ventilated at pressure settings unaltered from that delivered by the ventilator. Additional patients (n) added to the circuit are considered variable patients and can have their tidal volumes and PEEP altered by circuit components. This paper demonstrates use of a ventilator splitter adjusting for 1 control and *n* = 1 variable patients. The 1 + n(1) split contains Vent-Lock 3DP parts and commercial parts (Fig. 1). We 3D printed the splitters and the flow restrictors (needle valves). The other parts including the one-way check valves, the filters and the PEEP valves were commercial parts (Fig. S1). The Vent-Lock circuit was designed to be closed-circuit and leak-free to minimize risk of aerosolizing viral particles into the surrounding environment. The STL print files of these components are available as Data S1.

### Vent-Lock 3DP flow restrictor (FloRest)

The Vent-Lock 3DP flow restrictor (Vent-Lock FloRest) is a flow restrictor based on a needle valve design optimized for low flow rates to offer clinicians robust control over a range appropriate for human ventilation. Vent-Lock FloRest was designed to address the following concerns regarding ventilator “splitting” [2]: 1. Volumes would distribute unevenly between patients, 2. PEEP would be difficult to manage per patient, 3. Tidal volumes would be difficult to manage per patient, and 4. Adjustment or discontinuation of ventilation to one patient would alter breathing dynamics to other patients.

**Fig. 2:**
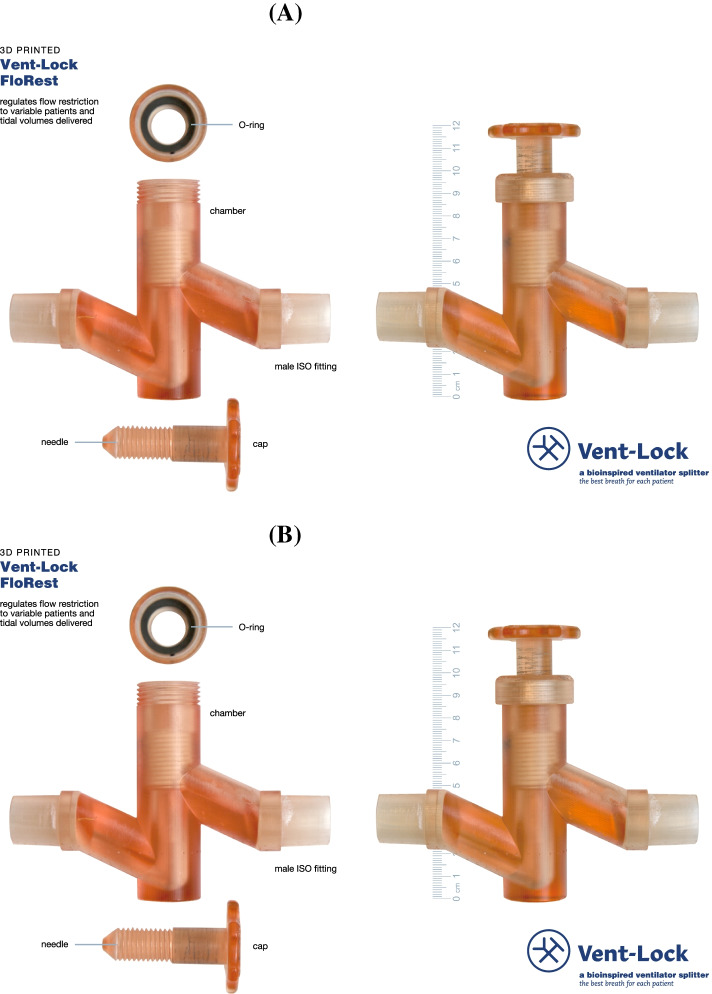
Design and performance of 3DP Flow Restrictor (FloRest). **A** Vent-Lock 3DP Flow Restrictor (FloRest) contains three components. The O-ring between the cap and full-height threaded needle interface at the top of the chamber to maintain air-tight seals. Both ends of Vent-Lock FloRest are male ISO fittings to ensure connection to ventilator tubing. **B** Testing with a ventilator on pressure control using simulation lungs at varying compliances demonstrates that Vent-Lock FloRest provides more control options than commercial ball valves and gate valves, characterized by more available data points corresponding with different tidal volumes delivered

The goal of FloRest (Fig. 2A) was to allow the clinician to modify the airway resistance delivered to the patient, thus providing ranges of flow rates, clinical tidal volumes, and PEEPs with control sensitivity and a reliable relationship between closure and flow rate, tidal volume, and pressures. The design emphasizes the minimization of build time and volume by reducing support material use and complex structures for consistent and higher quality printing. These considerations allowed for an air-tight and leak-proof design (Fig. S2) and utilization of biocompatible materials that can withstand extended exposure to warm humidified air and sterilizing autoclave environment. Using a particle counter, post and pre-autoclave tests demonstrate significant microparticle reduction after autoclaving (Fig. S3).

The needle valve utilizes change in flow momentum, flow path geometry and orifice flow design concepts allowing easy control of flow rate vs pressure drop ratio (i.e. flow coefficient) compared to gate and ball valve concepts (more binary valve concepts). The threading allows for control over the flow rates and offers the clinician the ability to make fine adjustments to the flow within the range of control. A gasket featuring an O-ring (E1000–212/AS568–212, O-Rings EPDM, FDA EPDM, Marco Rubber & Plastics, Seabrook, New Hampshire, USA) seated between the needle and chamber to ensure airtightness, thus reducing the risk of aerosolizing the virus into the surrounding environment. Final features of FloRest (Fig. 2A) included sealing to the external environment using unthreaded upper needle shafts for smoother interfaces between cap O-ring and needle during operation of valve and an increase in needle length prior to engagement of needle threading to provide more precise control of range of flow, and to allow for safe operation of valve by preventing full occlusion of flow to patient by clinician.

The FloRest designed with the clinician in mind, has advantages compared with commercial valves in terms of controllability, biocompatibility, and sterilizability. While FloRest had similar range of control compared to commercial gate valves (#P20034 PVC SCH 40 ½-in FNPT Ball Valve; G300 Lead Free Brass Gate Valve, American Valve, Greensboro, North Carolina, USA) (Fig. 2B). It was easier to control than commercial valves, characterized by more points available for the clinician to choose from corresponding to different tidal volumes delivered. Furthermore, FloRest is produced with biocompatible, nontoxic materials that can be safely sterilized, as compared to commercial ball valves with untested biocompatibility and unknown sterilization protocols. Vent-Lock FloRest can be produced at an estimated $3.50 per device in 3-hour 40-min print and process time via material extrusion (e3d, BigBox3D Ltd., Oxfordshire, UK; Little Monster, Tevo 3D Electronic Technology Co. Ltd., Zhanjiang, China) using PETG (PETG 3D Printer Filament, FilaMatrix, Virginia, USA). With vat photopolymerization (Form 2, Form 3, or Form 3B, Formlabs, Somerville, MA), it costs approximately $25, and 16 hours production time with a 50 μm build layer height resolution, using surgical guide resin (Surgical Guide, Formlabs, Somerville, MA). We demonstrate that the FloRest is leak-proof through air volume testing (Fig. S2), and leaky bubble test (Mov S1).

### Vent-Lock 3DP flow restrictor (FloRest) control of tidal volumes and PEEP

We tested the use of Vent-Lock FloRest in the Johns Hopkins Medicine Simulation Center (JHMSC) to confirm the following: 1) Allowing volumes to be distributed evenly between patients, 2) variable patient control of PEEP, 3) variable patient tidal volume control and 4) changes in the variable patient breathing settings does not alter breathing dynamics to the standard patient.

**Fig. 3:**
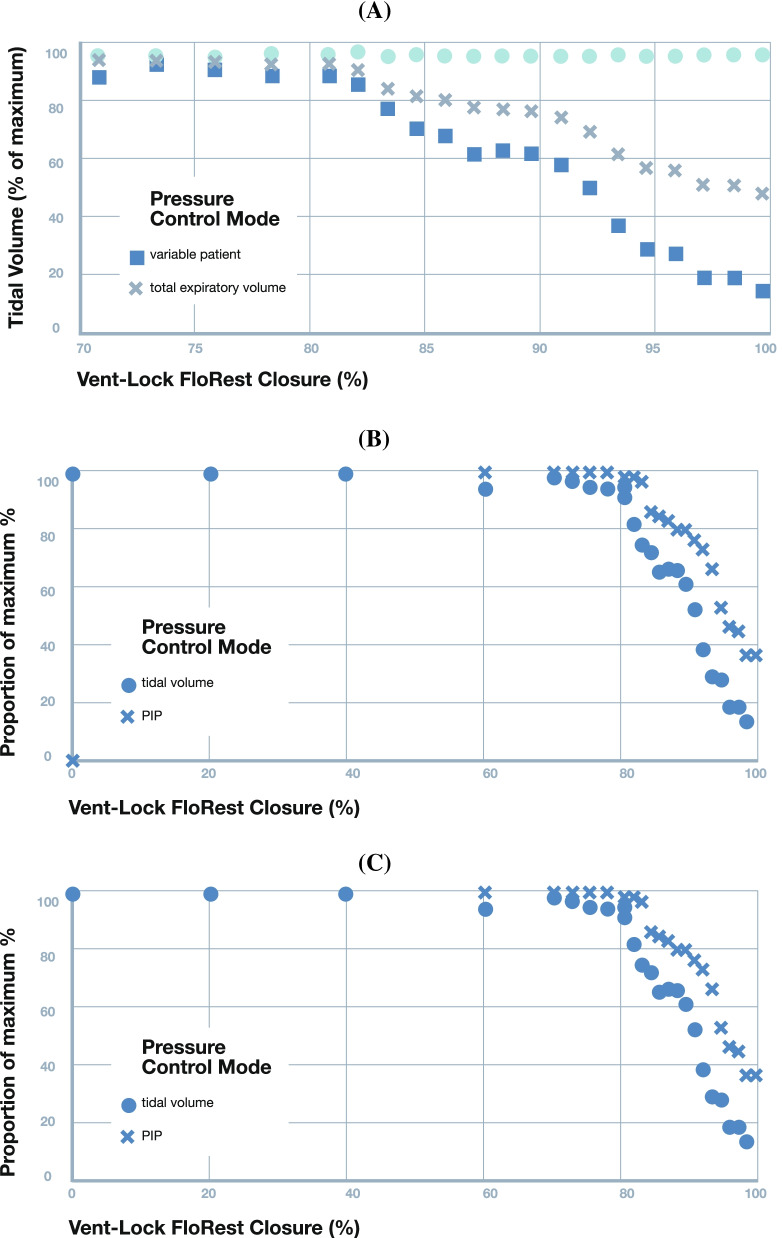
Pressure control mode: testing of Vent-Lock ventilator multiplexor with a ventilator on pressure control mode ventilating two simulated patients with different lung compliances. **A** When used with a ventilator on pressure control, Vent-Lock FloRest is capable of controlling tidal volumes delivered to the variable patient per turn, with no change to tidal volumes delivered to the standard patient. **B** PIP of the standard patient remains stable, despite changing PIP of the variable patient. Using a ventilator on pressure control, we determine the changes in standard and variable patient breathing pressures with closure of the Vent-Lock FloRest, and the ventilator reported pressures. The positive inspiratory pressure (PIP) of the variable patient decreases with closure, while the standard patient PIP, and ventilator reported average breathing pressures (P_avg_), and PEEP remain constant. We do note an increase in the ventilator reported PIP. **C** With the ventilator on pressure control, changes to tidal volume delivered to patients using Vent-Lock FloRest demonstrates a corresponding change in peak inspiratory pressures (PIP)

We tested the Vent-Lock multiplexing system using a 1 + 1 split patient circuit (Fig. 1). We used one ventilator (Puritan Bennett 840 Ventilator System, Avante Health Solutions) to ventilate two patients with different lung compliances of 20 mL/cmH_2_O and 50 mL/cmH_2_O, and monitored the intrapulmonary gas volumes, pressures, and compliances of the simulated patient lungs. We first tested using a pressure control mode, with inspiratory pressures set at 25 mL/cmH_2_O (additional ventilator settings available in Fig. S7). The Vent-Lock FloRest allowed adjustment of tidal volumes delivered to patients between 7- $\frac{3}{4}$ turns to 9- $\frac{7}{8}$ turns (fully closed); thus, the range of control for FloRest corresponds with $2-1/8$ turns (Fig. 3A). Within these turns, the tidal volume of the variable patient can be decreased by 85.7% (compared to initial variable patient tidal volume) with negligible change in tidal volume delivery to the standard patient (range: 99.86% and 103.2% initial standard patient tidal volume, mean: 102.1% ± 0.98%). We note that the total expiratory volume reported by the ventilator trends with tidal volume delivered to the variable patient (Fig. 3A) and the peak inspiratory pressures (PIP) of the variable patient and ventilator peak inspiratory volumes also correspondingly decrease with decreases in tidal volume (Fig. 3B, C), while peak inspiratory volumes remain stable for the standard patient and peak end expiratory volumes remain stable for both patients during these changes. Comparisons of Vent-Lock FloRest performances depending on materials for fabrication is available in Fig. S5.

**Fig. 4:**
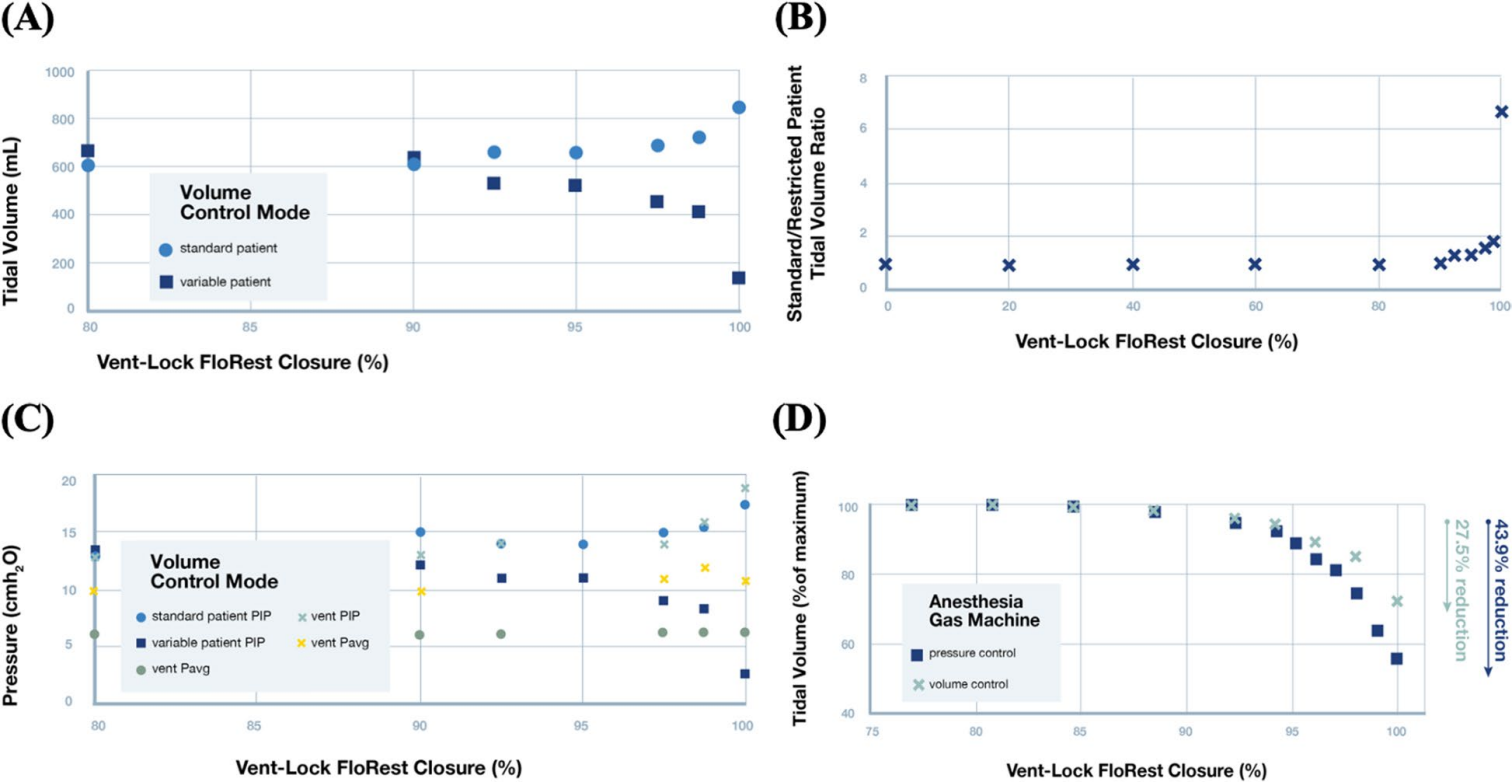
Volume control mode: testing of Vent-Lock ventilator multiplexer with a ventilator or anesthesia gas machine on volume control mode ventilating two simulated patients with different lung compliances. **A** With the ventilator on volume control, decreases in variable patient tidal volumes result in increases in tidal volumes delivered to the standard patient. This indicates that in volume control mode, patient ventilation circuits are interdependent, and changes in one patient effects the other. **B** The ratios of the patient’s tidal volumes (standard patient/variable patient) per closure of the Vent-Lock FloRest with ventilators on volume control. **C** On ventilator volume control and with Vent-Lock FloRest closure, the changes in peak inspiratory pressure (PIP) of the standard patient and variable patient reflect that of tidal volume changes, while the ventilator reported PIP and P_avg_ increase, and PEEP remains stable. **D** Vent-Lock FloRest was tested at Washington University in St. Louis using anesthesia gas machines. The FloRest can be used to control delivered tidal volumes to the variable patient on both pressure and volume control on anesthesia gas machines

We repeated Vent-Lock 1 + 1 multiplexing patient circuit with the ventilator on volume control mode to deliver a total of 2 L of volume, corresponding to approximately 600 mL of tidal volume per patient (additional ventilator settings available in Fig. S6). We note that turning of FloRest on the variable patient resulted in decrease of both tidal volume and PIP (Fig. 4A, B). However, this was accompanied with an increase in tidal volume delivery and PIP to the standard patient (Fig. 4A, B), with relatively stable ventilator reported average pressures (Vent P_avg_) and PEEP (Fig. 4B). Thus, unlike in pressure control mode where control of delivery to the variable patient was independent of the standard patient, flow restriction in the volume control mode resulted in the modification of the ratio of tidal volumes delivered (Fig. 4C, standard/variable patient tidal volume ratios). The optimized Ventilator settings on the 840 Ventilator System, Nellcor Puritan Bennett for these findings are avialble in Fig. S7.

We replicated results using anesthesia gas machines (North American Drager Narkomed 2a, Ardus Medical; GE Aestiva 57,900, Datex Ohmeda) at an alternate test site (Washington University in St. Louis, St. Louis, Missouri, USA) to demonstrate generalizability across locations and different resource settings. The 1 + 1 circuit was tested with the Vent-Lock FloRest on the variable patient. On both pressure control and volume control settings, the Vent-Lock FloRest demonstrated control of tidal volume delivered to the variable patient with stable tidal volumes delivered to the standard patient. Pressure control allowed slightly greater range of control (Fig. 4D, reduction of 43.9% tidal volume at close, compared to 27.5% reduction of tidal volume at close with volume control). These results indicate that both patients’ inspiratory and expiratory times, respiratory rates, and PEEP is determined by the ventilator or anesthesia gas machine’s settings. Changes in one patient’s condition does not alter these settings for either patient, as this is pre-determined by the ventilator settings. The variable that can be moderated during pressure-control setting is the tidal volume. The tidal volume is set a constant volume, and can be adjusted by Vent-Lock FloRest to optimize tidal volume to the variable patient, with no change to the constant patient. Therefore, this allows for constant, stable patient conditions, even while the clinician is optimizing tidal volumes to one of the patients.

### Real-time pressure reporting with Vent-Lock manometer adaptors

**Fig. 5:**
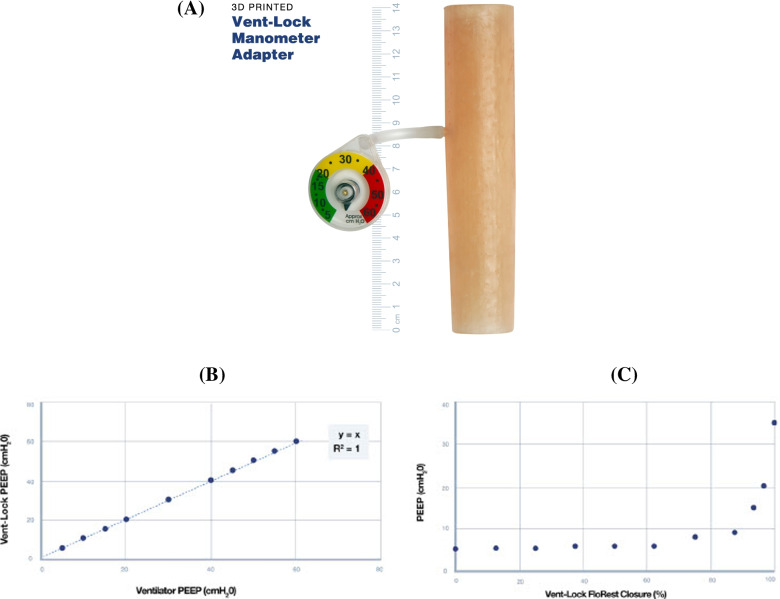
Vent-Lock manometer adaptor. **A** The Vent-Lock manometer adaptor is incorporated in the split circuit, and allows for the attachment of disposable manometers, thus provides accurate, real time readings of pressures. **B** When placed on the expiratory limb, the Vent-Lock manometer adaptor accurately reflects PEEP as set by the ventilator and as reported by the ventilator. **C** We use the Vent-Lock manometer to report the PEEP of the variable patient, as adjusted by the Vent-Lock FloRest on the expiratory limb. With closure of the FloRest, the PEEP increases

To facilitate continuous monitoring of pressures we designed a manometer adaptor that allows clinicians to either spot-check pressures or continuously monitor with the use of standard, disposable manometers such as those found on bag-valve-masks. The manometer adaptor can be added in the circuit at any point and is designed to accurately reflect breathing pressures, such as PIP and PEEP. We demonstrate that the manometer accurately reflects real-time pressures when incorporated in the circuit (Fig. 5A). In a 1 + 1 circuit with the ventilator on pressure control, the PEEP setting was incrementally increased, and associated ventilator detected PEEPs and Vent-Lock manometer reported pressures were recorded. The Vent-Lock manometer reported pressures were equivalent to the PEEPs (Fig. 5B). We also conducted blind tests, where one researcher set the PEEP on the ventilator and a second researcher (blinded to ventilator PEEP settings) reported PEEP as reported by the Vent-Lock manometer. The second researcher consistently and accurately reported all test values between 0 cmH_2_O to 50 cmH_2_O, in 5 cmH_2_O increments, with total ten trials with no error.

### Vent-Lock 3DP flow restrictor (FloRest) in swine

**Fig. 6:**
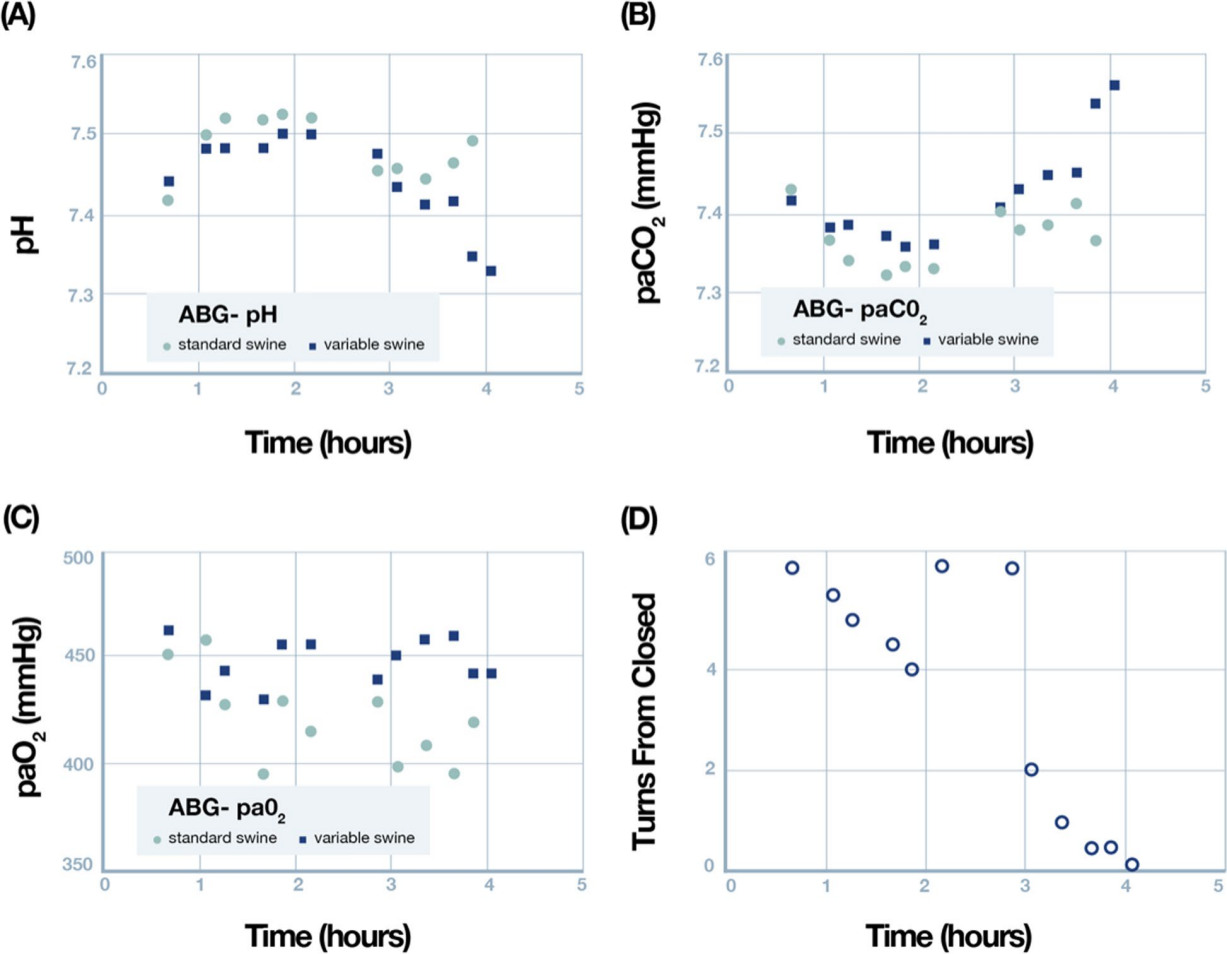
Serial Arterial Blood Gases were monitored throughout the experiment. **A** pH, **B** paCO_2_, and **C** paO_2_ are all plotted as a function of time. **D** Shows the number of turns from closed (with 6 turns being fully open) as a function of the time of the experiment. Note that the Vent-Lock system was reopened at approximately 2 hours due to development of hypercarbic alkalosis

**Fig. 7:**
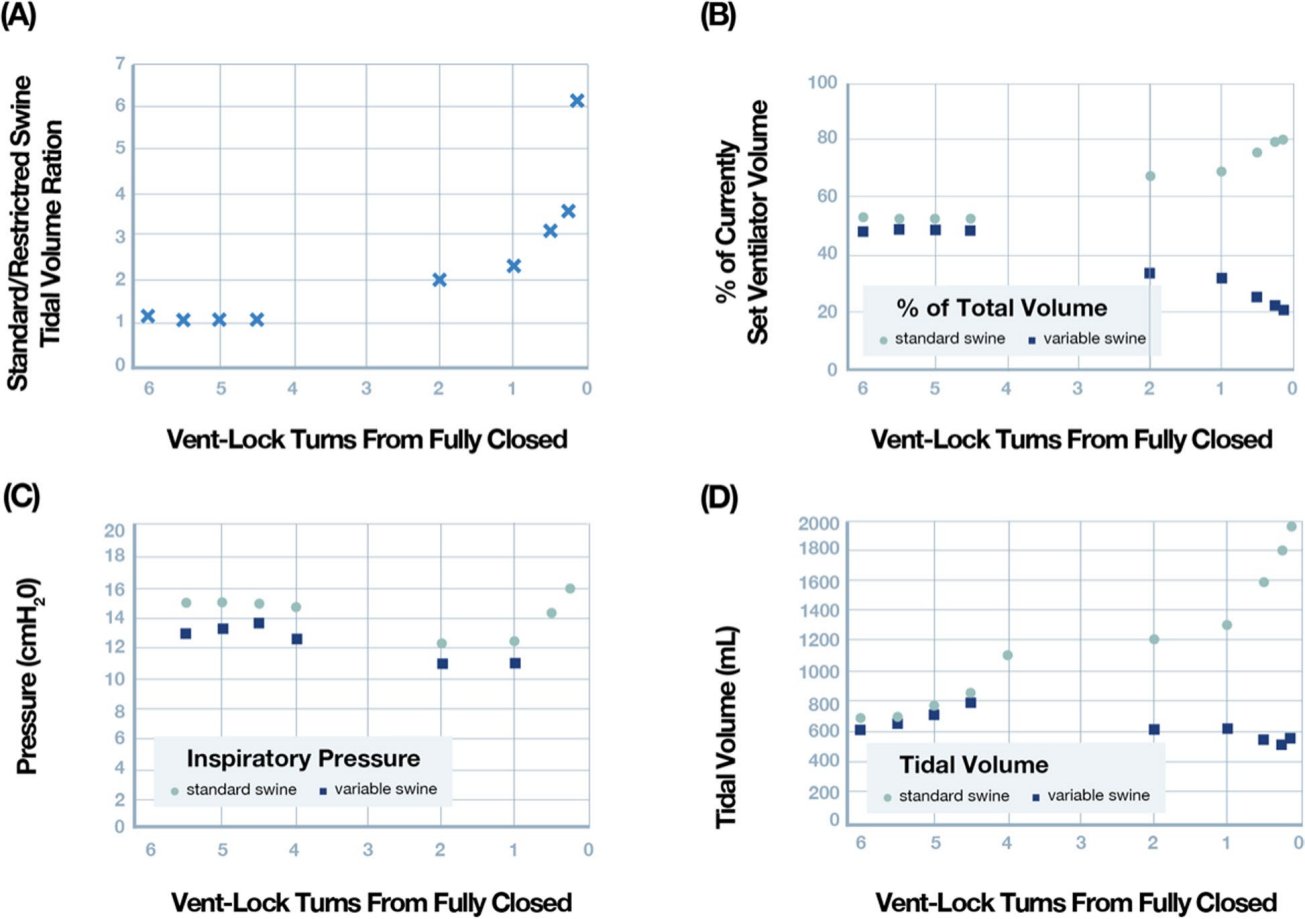
Ventilatory parameters as a function of Vent-Lock aperture with 6 turns indicating fully open and zero fully closed. The lowest turn plotted is 0.25 turns from fully closed. **A** Ratio of tidal volumes between the standard and variable swine. **B** Percent of currently set ventilator tidal volume measured in each swine. **C** Peak inspiratory pressure measured in each swine. **D** Tidal volume measured in each swine

Two domestic swine were anesthetized and ventilated using the Vent-Lock system with constant volume delivery. Swine were successfully ventilated for approximately 4 h using a single ventilator. Initial calculated dynamic lung compliances were 50.3 and 48.1 mL/cm H_2_O for the standard and variable swine, respectively. Throughout the experiment, minimum and maximum dynamic compliance ranged from 50.3 to 244.5 and 37.9 to 87.1 mL/cm H_2_O for the standard and variable swine, respectively, reflecting the differences in tidal volumes those swine received during the flow restriction trial. Serial ABGs were monitored (Fig. 6) and initial shared ventilator settings were determined to be too high as both swine developed a respiratory alkalosis. At approximately 2 h this was corrected and pH and paCO_2_ were allowed to normalize for 1 h. Vent settings were not changed following this equilibration. Over the next hour the Vent-Lock system was adjusted from fully open to fully closed, where air was still allowed to pass even when Vent-Lock is closed to prevent unintentional hypoventilation. Respiratory characteristics including tidal volume ratios, percent of total set tidal volume delivered, inspiratory pressure and tidal volume are presented in Fig. 7. While the tidal volume delivered to the variable swine decreased marginally, a substantial increase in tidal volume was noted to the standard swine (Fig. 7D), similar to what is seen in simulation center testing with ventilator on volume control mode. Arterial blood gas measurements demonstrated hyperoxia in both swine (Fig. 7C). A hypercarbic respiratory acidosis occurred in the variable swine (Fig. 6A, B) as the Vent-Lock closure reached its final turn. Necropsy performed to assess for gross lung pathology showed no significant findings of all lung lobes.

## Discussion

In the face of the COVID-19 pandemic, the importance of ventilators in the treatment of COVID-19 patients and the tenuous global supply of chain has resulted in the urgent need to increase ventilator capacity. One such solution to lack of ventilators is the ability to use one ventilator or anesthesia gas machine to support multiple patients. Previously reported clinical challenges include inability to independently control flow and pressure to each patient, development of a closed system to prevent viral contamination, and continuous monitoring. Additional engineering considerations identified in this manuscript includes designing for production in limited resource settings, material selection, clinician usability, and adaptability to various clinical settings and available supplies.

3D printing was selected as the prototyping and production modality due to rapid iterative production for research and development and on-demand manufacturing to meet urgent needs in context of biocompatible and sterilizable 3D printing materials. Our multi-disciplinary team of engineers and clinicians identified clinical, design, and materials challenges in prototyping.

Important design challenges for device production considerations include adaptability to all clinical indications for the device, associated risks with the device including additional risks due to the 3D printing process, FDA approved precedents to reduce the burden of pre-clinical testing, and ISO measurements for generalizability. Clinical indications and associated risks were identified through our multi-disciplinary team and guidelines from national societies. While no FDA approved precedents existed for ventilator splitters, we used ventilation parts (T-piece, incentive spirometers) to help guide designs. Additional risks due to the 3D printing process include design challenges resulting in inconsistency in production between different printer methods, make, and materials used, and suitable materials as detailed below:

- Design challenges for device production in limited resource settings include minimizing amount and number of materials used, time to print, minimizing post-processing, optimizing design to be compatible with a variety of printers and production methodologies, minimizing print fail points, minimizing number of parts, and fragility for transport (mechanical forces and temperature changes). 3D printed components of the splitter circuit were designed to include ventilator splitters, manometer adaptors for continuous pressure monitoring, and a flow restrictor to control tidal volumes and pressures, with these critical points kept in mind. The final design uses one material, requires minimal assembly and post-processing, and parts can be positioned so that they are printed in one print job. Our devices were successfully mailed between institutions without damage, and were still functional following mailing, demonstrating promise for distribution. These are critical design points that all multidisciplinary teams should consider when designing and producing devices for limited resource settings.
- Materials challenges vary by medical application. For ventilator associated parts, some example considerations include surface finish (smooth to decrease unintentional resistance), biocompatibility and chemical emissions (exposure to airway and airway secretions), sterilizability, reduction in health risks. Surgical guide resin (Surgical Guide, Formlabs, Somerville, MA) was chosen as the material for our circuit due to evidence of low chemical emissions from cured parts [37], and is noncytotoxic, non-irritating, and complies with ISO10993-1:2018 [33] biocompatibility guidelines. Additionally, it has a smooth surface finish, and can be sterilized without increasing chemical emissions. We demonstrated that the parts could be autoclaved or chemically disinfected (with EtOH) without device distortion, function, or leak. This is promising as autoclave, microwave disinfection, and chemical disinfection are more readily available in developing countries [38].
- Universal production: Production in limited resource settings requires minimizing number of materials used, minimal support structures that result in material waste, decrease in failure points, compatibility with different printers, and utilization of both vat photopolymerization and material extrusion. While the reproducibility of the Vent-Lock FloRest has yet to be rigorously investigated, the design itself was edited by our team of engineers to use one material, use minimal support structures, and decrease fragile angles that result in failure. The design has been successfully produced from a variety of 3D printers of either material extrusion or vat photopolymerization. We recommend five key considerations (resolution, surface quality, biocompatibility, sterilizability, and tolerancing) to successfully utilize other methods and machines for universal production in Fig. S8. Vat photopolymerization is the preferred production modality as the layer resolution, liquid starting material, and curing process minimize microscale air gaps for bacterial seeding, and smoother finish [14]. However it should be noted that vat photopolymerization is not as commonly available as material extrusion, and thus production should be optimized for both methods, and vat photopolymerization only when medically indicated.

These challenges highlight the critical nature of a multidisciplinary team with both clinicians and engineers to discuss unbeknownst challenges, and formulate solutions. This organic relation results in rapid “ready for clinical use” designs that efficiently utilizes time, personnel, and materials in limited-resource settings, while reducing patient risks. This approach has allowed our team to produce a ventilator multiplexing circuit with considerable differences compared to existing works. For example, many innovative teams have successfully demonstrated the concept of a flow restrictor valve to provide tailored volume deliveries. However, these designs often do not provide precise control within a clinically significant range (e.g. repurposed commercially available medical or industry valves), large intervals of tidal volume set points making titration difficult (e.g. in-line check valves [39], Hoffman clamps [40]), or requires disruption of the circuit in order to moderate flow (e.g. series of tracheal tubes [40], flow limiters of varying sizes [41]). The FloRest is optimized to be clinician-friendly (optimization of threading and counts, easy to turn needle, ISO threading to adapt to most ventilator tubing) allowing for precise control by the clinician without having to disrupt the circuit. Many reported Y-splitter designs have harsh or arbitrarily chosen angles. In contrast, our splitter was bioinspired by the angle and curvature of the human bronchi, economically designed by nature to balance optimal and uniform air delivery into branching limbs, while minimizing associated pressure losses due to branching losses and Poiseuille losses [42, 43].

It is important to note that we consider the available resources in environments with the greatest need, and validated ventilation multiplexing using anesthesia gas machines. Anesthesia gas machines have ventilation functions, and are widely available globally, even in developing countries. They are well suited to be repurposed for ICU ventilation in the face of ventilator shortages, and considerations for modification and usage settings have been addressed [44]. This builds upon previous work on bench testing of anesthesia gas machine multiplexing (previously without tidal volume or airway pressure controls) [45], and demonstrates user regulation in large animal trials (Fig. 4D). Therefore, our study is the highest evidence to-date that anesthesia gas machines can be modified with external circuits (i.e. Vent-Lock 3DP circuit) to provide anesthesia gas machines with more ventilation controls for multiplexing.

These engineering considerations may help minimize the risks of medical devices due to the 3D printing process compared to traditional manufacturing precedents. However, engineering optimization may not address the inherent medical risks of the device or change its indications. Our ventilator circuit was designed to address the concerns raised by the SCCM on ventilator splitting. The successful ventilator splitting with Vent-Lock mitigates, but does not eliminate concerns [46]. For example, one concern was the setting (pressure vs. volume control) of the ventilator. Our studies verify the fundamental differences in tidal volume patterns with flow restriction with the ventilator in volume versus pressure mode, and how to address this on both legacy ventilators and anesthesia gas machines. In pressure control, Vent-Lock FloRest allowed for reduction in delivered tidal volumes to the variable patient with stable volumes delivered to the standard patient (Fig. 3A). However, in volume control, reduction in delivered tidal volume to the variable patient resulted in a concomitant increase in tidal volume delivery to the standard patient. This pattern is expected due to the continuous delivered volume maintained by the machine; therefore, the Vent-Lock FloRest allowed regulation of the standard/variable patient tidal volume ratio as expected for conservation of fluid flow following the principle of continuity (Fig. 4B). This pattern of ratio control is seen in both ventilator volume control settings in the simulation center and replicated in vivo swine studies using anesthesia gas machines (Fig. 5). The variation in both patients may be difficult for clinicians to manage simultaneously. However, this ratio-based control delivered by Vent-Lock FloRest can be critical for splitting legacy ventilators or anesthesia gas machines that may only have volume control settings.

One of the biggest challenges of splitting patients on ventilators is that air will preferentially travel to the patient with the highest baseline lung compliance resulting in unequal ventilation between the two patients. However, if Vent-Lock FloRest is placed on the patient with the highest baseline lung compliance (variable patient), air flow can be decreased, while simultaneously increasing air flow to the standard patient, until tidal volumes are equilibrated between the two. Similar investigations have also found that ventilator splitting requires delicate titration of air flow and pressures, but resulting fluctuations in the standard patient may be manageable [47]. One group found that despite these nuances requiring clinician adjustments, ARDS porcine models on multi-porcine ventilators had similar oxygenation and morbidity compared to ARDS single-ventilated porcine [48]. Thus, we emphasize that especially during emergency use settings, providers appreciate these differences in tidal volume control mechanisms, and select the settings most appropriate for patients and their changing clinical statuses. We consider that ventilator multiplexing is an alternative but should not be mistaken as a solution that may potentially provide a dangerous false sense of safety.

When the FloRest is placed on the expiratory loop of the patient, restriction of airflow results in pressure increases between the patient and the FloRest, effectively functioning as a PEEP valve (Fig. 5C), which can then be reported by the manometer and adapter. This PEEP change established by FloRest and continuous monitoring does not affect the other patient split on the ventilator. Therefore, this is a critical asset of the circuit that allows for more patient-tailored PEEP therapy which is especially important in the treatment of patients with ARDS due to COVID-19 or other lung pathologies. While most PEEP valves currently rely on a spring-loaded control system, this may be difficult to produce rapidly, especially via additive manufacturing. Our design demonstrates control of expiratory pressures through flow restriction. However, we do note challenges with FloRest in creating PEEP control, including that the PEEP was not changed until near complete occlusion of the valve, at which point additional turns resulted in rapid changes in PEEP (Fig. 5C). Consequently, we recognize that Vent-Lock FloRest requires further optimization and testing prior to clinical usage but exists as a proof-of-concept that PEEP control may be possible through a spring-less system, which would be more readily available in limited-resource settings.

Some limitations to our study include lack of human clinical testing. While the swine in this study had lung compliances like humans, it is unknown how well this reflects human physiology. Therefore, results may not be translatable due to species anatomy and clinical scenario differences. Due to this, we must also consider the hypothetical limitations of multiplexing. For example, while it may be theoretically possible to multiplex three or more patients on one ventilator using computer modeling [49, 50], or lung models [23, 51], this may not be feasible nor safe in practice due to circuit complexity resulting in potential confusion and cross contamination, overburdening of the ventilator’s capacity, and other unknowns. Challenges in continuous monitoring must be addressed prior to human studies to ensure patient safety. Future directions include developing a more rigorous continuous monitoring of flow rates and delivered tidal volumes to patients to facilitate adjustments of flow per FloRest. This is critical due to the dynamic lung physiologies of patients with ARDS and preventing barotrauma or under or over ventilation. Therefore, we recommend setting a target lung volume per patient, and monitoring via spirometry or airflow transducers, such as the ones used in our swine studies (SS11LB airflow transducer (Biopac; Goleta, CA)). Patient lung volumes and their oxygenation statuses should be spot checked with the spirometer or transducers and arterial blood gases. Lastly, we emphasize that ventilator multiplexing is only to be used in emergency situations after all alternatives have been exhausted. Despite our findings of improved ventilator multiplexing function through engineering optimization, additional studies are required to validate the safety and clinical considerations in translation to human subjects. However, as future pandemics and disasters may exhaust standard-of-care for patient ventilation, Vent-Lock and the multidisciplinary approach to assess both clinical and engineering challenges may be useful to develop future solution if “the other option is death” [52].

During the COVID-19 pandemic, open sourcing has been the fuel to a burst of ingenuity by engineers and clinicians globally to aid their communities [53]. Open sourcing and 3D printing have been proven to be helpful in the developing world by providing low cost, easy to use medical products, low cost construction of homes, water treatment devices and prosthetic limbs [54]. Thus, utilization of 3D printing to produce Vent-Lock circuit and Vent-Lock FloRest allows for the rapid, on-demand, on-site production to meet immediate needs. However, we recognize that our recommendations and standards are specific for the materials, printers and sterilization protocols reported in our methodologies. Therefore, our specific protocols can limit production in developing world with limited access to these supplies and materials. While we provide parameters for resolution and strength for selection of other materials, further tests should be performed in varying methodologies and materials to ensure accuracy in the printing process and translation into actual use. While it is promising that our materials have remained stable in humidified 40 °C for over 48 hours (Fig. S3), further testing is required to ascertain stability across pressure gradients over weeks to months of use, and in a diversity of environments.

Additionally, we do recognize the limitations of additive manufacturing. The value of Vent-Lock circuit is its ability to be stored in preparation of emergency situations, such as disaster preparedness or in military combat zones, where ventilator shortages can be expected. In this case, we believe that while 3D printing production can meet initial interests, traditional manufacturing (such as injection molding), may be a more cost-effective and time-efficient approach to fulfill demand. However, it is important to note that optimization of designs for 3D printing is very different than for manufacturing. For example, 3D printing allows for very fine details like printing personalized text on a surface – something that cannot be simply achieved via manufacturing.

## Conclusions

In this study, we developed Vent-Lock, a ventilator or anesthesia gas machine splitter system with a flow restrictor (FloRest) that can modify flow rates per patient for patient-tailored therapies. We provide proof-of-concept that two swine can be ventilated using one anesthesia gas machine. While additional work is critical for the safe use of ventilator multiplexing, our experiences reiterate the clinical challenges, and introduce the engineering practicalities of translational design one must consider while designing medical devices for limited resource settings. Not only must the device function, but it must function reliably within a diversity of environments, and adaptable to the intricacies of the human body and pathologies.

## Supplementary Information

**Additional file 1: Fig. S1.** De novo ventilator circuit components produced via 3D printing.

**Additional file 2: Fig. S2.** Air-tightness tests of the Vent-Lock FloRest.

**Additional file 3: Fig. S3.** Design files for Vent-Lock splitters, needle valve, and manometer adaptor.

**Additional file 4: Fig. S4.** Tests of tidal volume control with and without O-rings.

**Additional file 5: Fig. S5.** Comparisons of Vent-Lock FloRest performances depending on materials.

**Additional file 6: Fig. S6.** The biodurability and sterilization conditions.

**Additional file 7: Fig. S7.** Ventilator settings on 840 Ventilator System, Nellcor Puritan Bennett.

**Additional file 8: Fig. S8.** Five key requirements (resolution, surface quality, biocompatibility, sterilizability, and tolerancing) for methodologies to successfully utilize other methods and machines for parts production.

**Additional file 9: Mov S1.** Leaky bubble test demonstrates Vent-Lock FloRest is airtight.

**Additional file 10: Data S1.** STL print files of Vent-Lock splitter, FloRest, and manometer adaptor.

**Additional file 11: Data S2.** MATLAB code used to analyze swine data.

## Data Availability

The data supporting the findings of this study is available within the article and its Supplementary material. Additional materials are available upon reasonable request from the corresponding authors.

## Abbreviations

3DP, 3D printing: Three-Dimensional Printing
ARDS: Acute Respiratory Distress Syndrome
COVID-19: Coronavirus disease 2019
FloRest: Flow restrictor
FDM: Fused deposition modeling
G code: Geometric code
ICU: Intensive Care Unit
IPA: Isopropyl alcohol
PEEP: Positive end expiratory pressure
PIP: Positive inspiratory pressure
SARS-CoV-2: Severe acute respiratory syndrome coronavirus 2
SCCM: Society of Critical Care Medicine
SLA: Stereolithography
USA: United States of America
