# Supplementary material for: Translational design for limited resource settings as demonstrated by Vent-Lock, a 3D-printed ventilator multiplexer
Source: 3D Print Med. 2022 Sep 14;8:29. doi: 10.1186/s41205-022-00148-6 (PMC9471031; doi:10.1186/s41205-022-00148-6)
Supplement: Supplementary file 1 — Additional file 1: Fig. S1. De novo ventilator circuit components produced via 3D printing. [file 41205_2022_148_MOESM1_ESM.pdf]

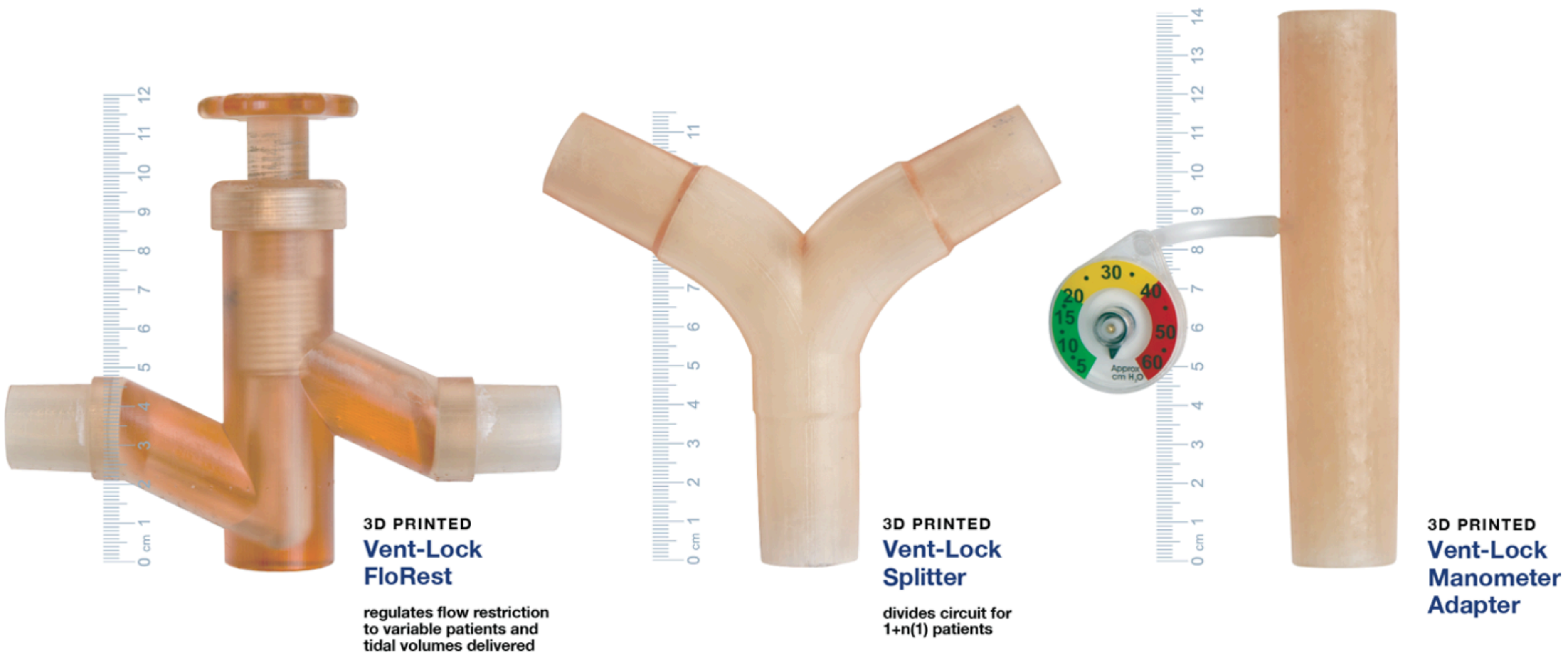

Supplementary Figure 1. *De novo* ventilator circuit components produced via 3D printing.

The Vent-Lock ventilator splitter circuit features *de novo* ventilator circuit components produced via 3D printing. All components have ISO standard sizing to allow for fitting with universal ventilators, anesthesia gas machines, and associated tubing. We picture here the ventilator splitter (left), FloRest (center), and manometer adaptor (right) with example of manometer attached.
