# Supplementary material for: Translational design for limited resource settings as demonstrated by Vent-Lock, a 3D-printed ventilator multiplexer
Source: 3D Print Med. 2022 Sep 14;8:29. doi: 10.1186/s41205-022-00148-6 (PMC9471031; doi:10.1186/s41205-022-00148-6)
Supplement: Supplementary file 2 — Additional file 2: Fig. S2. Air-tightness tests of the Vent-Lock FloRest. [file 41205_2022_148_MOESM2_ESM.pdf]

(A)

Ventilator on volume control, delivering 500 mL	tidal volume average	tidal volume st dev
tubing	366.8	2.38
tubing + Vent-Lock FloRest (open)	368.6	1.34
tubing + Vent-Lock FloRest (closed)	367.2	0.45

(B)

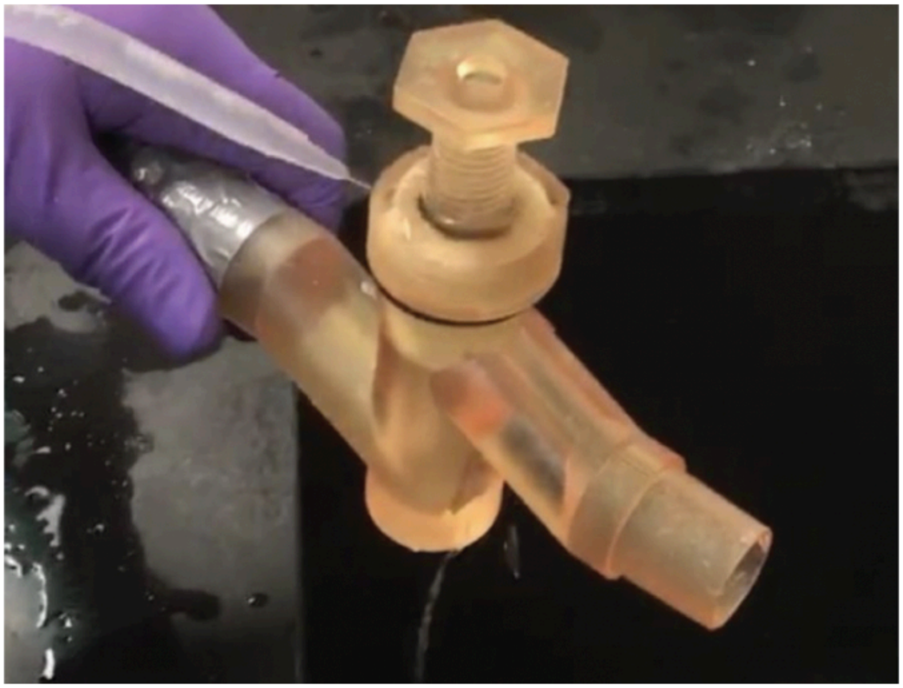

Supplementary Figure 2. Air-tightness tests of the Vent-Lock FloRest. (A) We demonstrate that Vent-Lock FloRest is air-tight by comparing total delivered tidal volumes to simulated patient lungs with standard tubing (control), compared to with the FloRest in the circuit fully open, or fully closed. The comparable volume delivered to patient lungs demonstrates minimal air leakage across the FloRest. (B) This is further confirmed by soapy bubble testing (**Mov S1**) where FloRest is covered in soap water and attached to air; if there was air leakage across the FloRest, soap bubbles would emerge from the device. We demonstrate that there were no soap bubbles that emerged during soapy bubble testing.
