# Supplementary material for: Translational design for limited resource settings as demonstrated by Vent-Lock, a 3D-printed ventilator multiplexer
Source: 3D Print Med. 2022 Sep 14;8:29. doi: 10.1186/s41205-022-00148-6 (PMC9471031; doi:10.1186/s41205-022-00148-6)
Supplement: Supplementary file 4 — Additional file 4: Fig. S4. Tests of tidal volume control with and without O-rings. [file 41205_2022_148_MOESM4_ESM.pdf]

(A)

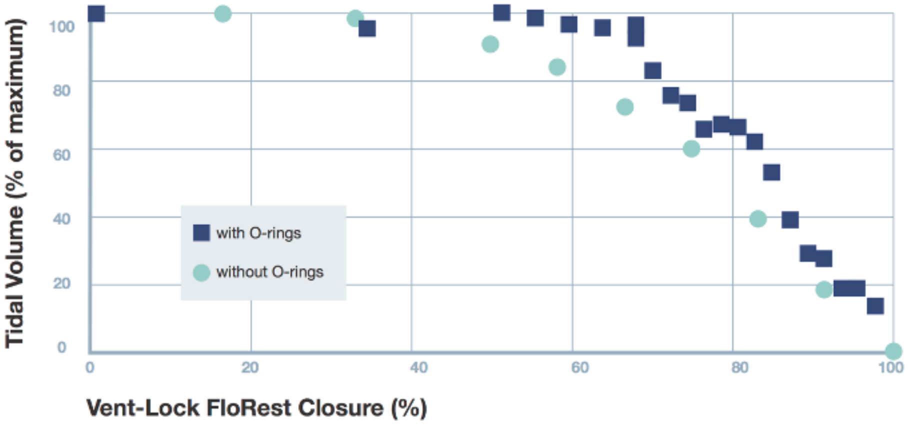

(B)

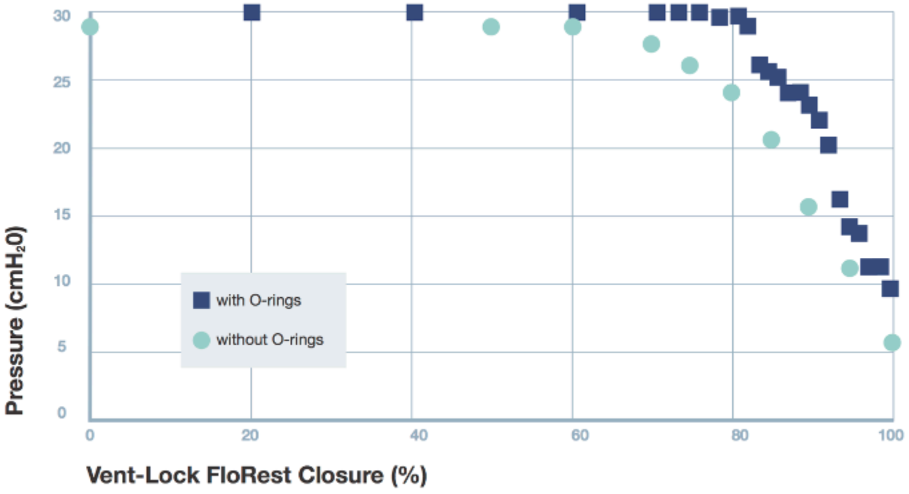

Supplementary Figure 4. Tests of tidal volume control with and without O-rings.

Vent-Lock FloRest is designed to seat O-rings, which create an airtight seal, thus reducing risk of releasing patient respiratory droplets into the patient room. There are differences in (A) tidal volume and (B) pressure control per turn of Vent-Lock with and without the O-rings.
