# Supplementary material for: Translational design for limited resource settings as demonstrated by Vent-Lock, a 3D-printed ventilator multiplexer
Source: 3D Print Med. 2022 Sep 14;8:29. doi: 10.1186/s41205-022-00148-6 (PMC9471031; doi:10.1186/s41205-022-00148-6)
Supplement: Supplementary file 5 — Additional file 5: Fig. S5. Comparisons of Vent-Lock FloRest performances depending on materials. [file 41205_2022_148_MOESM5_ESM.pdf]

(A)

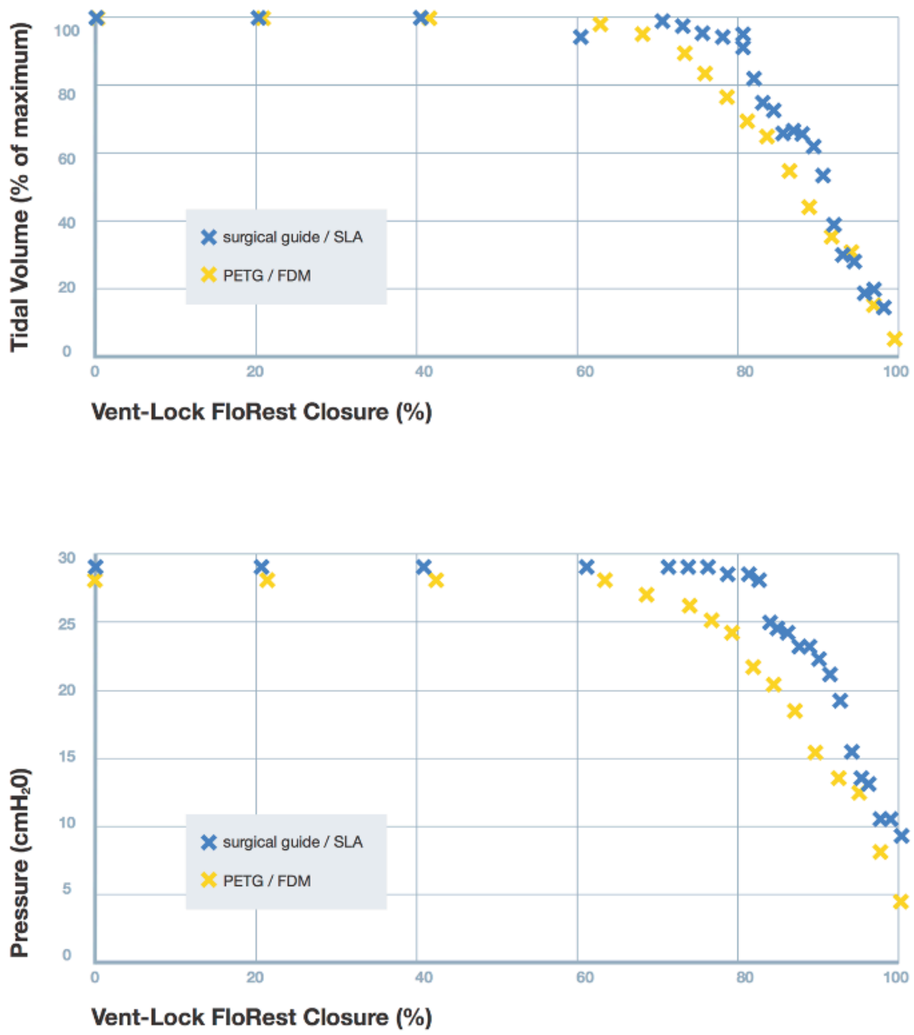

(B)

Supplementary Figure 5. Comparisons of Vent-Lock FloRest performances depending on materials. Vent-Lock FloRest has been produced with both Formlabs Surgical Guide resin via stereolithography, and PETG via FDM. The material option impacts the control of the (A) tidal volume or (B) pressure per turn of Vent-Lock.
