# Supplementary material for: Translational design for limited resource settings as demonstrated by Vent-Lock, a 3D-printed ventilator multiplexer
Source: 3D Print Med. 2022 Sep 14;8:29. doi: 10.1186/s41205-022-00148-6 (PMC9471031; doi:10.1186/s41205-022-00148-6)
Supplement: Supplementary file 6 — Additional file 6: Fig. S6. The biodurability and sterilization conditions. [file 41205_2022_148_MOESM6_ESM.pdf]

(A)

Biodurability and Sterilization Conditions								
particle size (microns)	HEPA filter	commercial ventilator tubing	surgical guide conduit		surgical guide conduit post-IPA sterilization		surgical guide conduit post-IPA sterilization 48-hour exposure to humidified hgeat	
			pre-autoclave	post-autoclave	pre-autoclave	post-autoclave	pre-autoclave	post-autoclave
0.3	3	3656	116	23	832	443	833	14
0.5	0	261	62	7	72	38	39	1
1	0	29	55	7	16	3	8	1
3	0	3	33	7	4	0	1	1
5	0	1	24	7	3	0	1	1
10	0	1	13	5	2	0	1	1

(B)

production	material	sterilization
FFF	PETC / PETG	70% isopropyl alcohol bath for five minutes<br>bleach bath<br>UV-VIs
SLA	dental surgical guide<br><br>surgical guide	Autoclave (3x 20 minute cycles, 250° F<br>alcohol bath<br>UV-VIs

Supplementary Table 1. The biodurability and sterilization conditions. (A) We test the biodurability of the surgical guide resin via particle count following autoclave, IPA sterilization, or exposure to humidified heat for 48 hours. We compare the particle counts to the gold standard HEPA filter, and to commercially available ventilator tubing. (B) We recommend the following sterilization conditions following recommended testing in lab and manufacturing recommendations.
