# Supplementary material for: Translational design for limited resource settings as demonstrated by Vent-Lock, a 3D-printed ventilator multiplexer
Source: 3D Print Med. 2022 Sep 14;8:29. doi: 10.1186/s41205-022-00148-6 (PMC9471031; doi:10.1186/s41205-022-00148-6)
Supplement: Supplementary file 7 — Additional file 7: Fig. S7. Ventilator settings on 840 Ventilator System, Nellcor Puritan Bennett. [file 41205_2022_148_MOESM7_ESM.pdf]

Ventilator and Ventilator Alarm Settings	
**ventilator parameter**	**value**
pressure (on pressure control mode)	25 cmH2O
volume (on volume control mode)	2L for 2 patients
PEEP	5 cmH2O
respiratory rate	8
I/E ratio	0.8
**ventilator alarm settings**	**value**
tidal volume	150 mL
pressures	60 to 70 cmH2O
breath trigger	-20 to -30 cmH2O

Supplementary Table 2. Ventilator and ventilator alarm settings on 840 Ventilator System, Nellcor Puritan Bennett. Ventilator settings used in testing the Vent-Lock ventilator multiplexing system in the simulation center and with swine studies. Alarm trigger settings on the ventilator; please note that alarm triggers are set to be at far extremes to prevent ventilator from terminating ventilation, or from one patient triggering a breath for the entire circuit.
