# Supplementary material for: Translational design for limited resource settings as demonstrated by Vent-Lock, a 3D-printed ventilator multiplexer
Source: 3D Print Med. 2022 Sep 14;8:29. doi: 10.1186/s41205-022-00148-6 (PMC9471031; doi:10.1186/s41205-022-00148-6)
Supplement: Supplementary file 8 — Additional file 8: Fig. S8. Five key requirements (resolution, surface quality, biocompatibility, sterilizability, and tolerancing) for methodologies to successfully utilize other methods and machines for parts production. [file 41205_2022_148_MOESM8_ESM.docx]

| **Requirements** | **Recommendations** | **Description & Rationale** |
| --- | --- | --- |
| 1. **Resolution** | LR > 0.2 mm | The first guideline is the highest possible resolution of the 3D printer; if the layer resolution (LR) is lower than 0.2 mm layer height, then the 3D printed part risks high porosity and permeability through the large layer line gaps. An increase in the resolution will lead to smaller gaps for air to escape. |
|  | SLA > FDM | To fully minimize air leaks, SLA printing is preferred rather than FDM printing as it prints solid, photopolymerized parts with up to 50 µm layer resolution. |
| 1. **Surface Quality** | FDM: regulated printing conditions | Surface quality is heavily affected by the type of 3D printing and material. FDM printing is at a larger risk for poor surface quality due to the nature of the process as temperature deviations can cause large variations from print to print and even between layers. If the nozzle temperature and environmental temperature are not consistent, FDM prints can warp, curl and string, leading to rough surface quality which requires additional post-processing such as sanding to remove defects. Printers with reliable heating and cooling through heated chambers and build plates mitigate this issue. |
|  | Supports:  FDM - water soluble  SLA - minimize touchpoints | Removing support scaffolding can also result in rough surface artifacts.   - For FDM, this can be prevented if water-soluble supports are used, in which the surface quality greatly improves. - SLA printing has a similar issue, tree-shaped supports may leave undesirable bumps or marks on the print. Decreasing the touchpoint size and sanding the surface post-processing will ensure the surface is smooth. As underlined previously, do not print supports within the internal cavities of the parts as it may affect the quality in these areas significantly. |
| 1. **Biocompatibility** | Robust pre-clinical testing   - Simulation center - Animal studies | Material selection regarding biocompatibility is important, as it should be non-irritating and non-toxic after it is 3D printed. Materials and devices must satisfy International Standards for biological evaluation of medical devices (ISO 10993). Devices must be tested to minimize risks over time and use of device, and with breakage potentially resulting in exposure of tissues to novel materials. |
| 1. **Sterilizability** | Dry Vacuum Autoclave  &  Chemical Sterilization | The material should be easily sterilizable with minimal integrity to the device structure and function. Sterilization methodologies must be reliable, flexible, and rapid for optimization for clinical use. The material should satisfy International Standards for healthcare products.  Dry Vacuum Autoclave (3 cycles at 120.0 ^o^C, 20 min sterilize, and 20 min dry time)  Chemical Sterilization (>99.5% isopropyl alcohol bath for 30 min, air-dried at 22 ^o^C for 30 min, and placed in an oven at 40 ^o^C in humidified air for 48 hours) |
| 1. **Tolerancing** | Tolerance  FDM: 0.1 to 0.2 mm  SLA: 0.05 to 0.1 mm | Tolerancing is dependent on the 3D printing method.  FDM prints require a larger tolerance between parts that interface with each other as the components undergo thermal expansion when printing, which if not accounted for, will cause these parts to not properly fit. For FDM prints, we implemented a tolerance of around 0.1 to 0.2 mm for multiple pieces.  For SLA printing, the tolerances can be smaller, as the parts will have more dimensional accuracy due to the higher resolution. For SLA the tolerances were half of FDM prints (0.05 to 0.1 mm for multiple pieces). |

**Fig. S8.** Five key requirements (resolution, surface quality, biocompatibility, sterilizability, and tolerancing) for methodologies to successfully utilize other methods and machines for parts production.

.
