# Supplementary material for: Translational design for limited resource settings as demonstrated by Vent-Lock, a 3D-printed ventilator multiplexer
Source: 3D Print Med. 2022 Sep 14;8:29. doi: 10.1186/s41205-022-00148-6 (PMC9471031; doi:10.1186/s41205-022-00148-6)
Supplement: Supplementary file 3 — Additional file 3: Fig. S3. Design files for Vent-Lock splitters, needle valve, and manometer adaptor. [file 41205_2022_148_MOESM3_ESM.pdf]

(A)

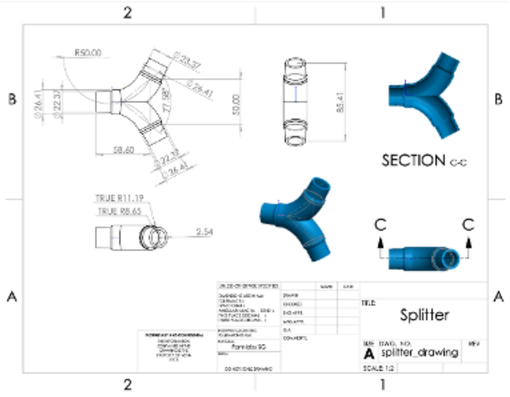

(B)

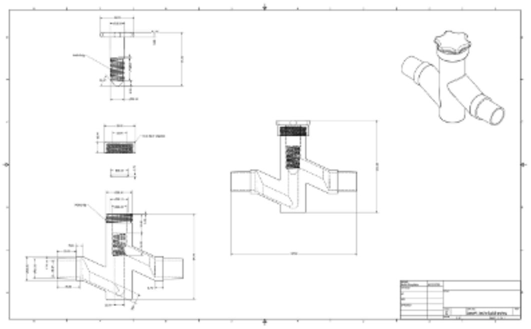

(C)

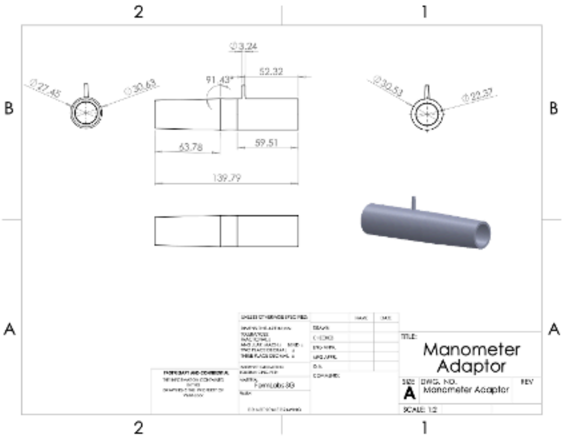

Supplementary Figure 3. Engineering drawings for Vent-Lock (A) splitters, (B) FloRest, and (C) manometer adaptor.
